# Mitochondrial fusion and Bid-mediated mitochondrial apoptosis are perturbed by alcohol with distinct dependence on its metabolism

**DOI:** 10.1038/s41419-018-1070-3

**Published:** 2018-10-09

**Authors:** Shamim Naghdi, William S Slovinsky, Muniswamy Madesh, Emanuel Rubin, György Hajnóczky

**Affiliations:** MitoCare Center, Department of Pathology, Anatomy and Cell Biology, Thomas Jefferson University, Philadelphia, PA USA

## Abstract

Environmental stressors like ethanol (EtOH) commonly target mitochondria to influence the cell’s fate. Recent literature supports that chronic EtOH exposure suppresses mitochondrial dynamics, central to quality control, and sensitizes mitochondrial permeability transition pore opening to promote cell death. EtOH-induced tissue injury is primarily attributed to its toxic metabolic products but alcoholism also impairs tissues that poorly metabolize EtOH. We embarked on studies to determine the respective roles of EtOH and its metabolites in mitochondrial fusion and tBid-induced mitochondrial apoptosis. We used HepG2 cells that do not metabolize EtOH and its engineered clone that expresses EtOH-metabolizing Cytochrome P450 E2 and alcohol dehydrogenase (VL-17A cells). We found that fusion impairment by prolonged EtOH exposure was prominent in VL-17A cells, probably owing to reactive oxygen species increase in the mitochondrial matrix. There was no change in fusion protein abundance, mitochondrial membrane potential or Ca^2+^ uptake. By contrast, prolonged EtOH exposure promoted tBid-induced outer mitochondrial membrane permeabilization and cell death only in HepG2 cells, owing to enhanced Bak oligomerization. Thus, mitochondrial fusion inhibition by EtOH is dependent on its metabolites, whereas sensitization to tBid-induced death is mediated by EtOH itself. This difference is of pathophysiological relevance because of the tissue-specific differences in EtOH metabolism.

## Introduction

Environmental stressors commonly rewire cellular signaling pathways resulting in either the cell’s demise or adaptation supporting survival. Many of these pathways converge on mitochondria, which provide energy and directly control cell survival and ion homeostasis. Mitochondrial dynamics is necessary to maintain mitochondria in optimal condition and mitochondrial membrane integrity is required to support cell survival.

Mitochondrial dynamics involves fusion, mediated by MFN1/2^1^ and OPA1^2^ and fission, mediated by DRP1 with the assistance of MFF, MID49/51, dynamin 2, and perhaps FIS1^3^. Fusion mediates the exchange of mtDNA, proteins, and other soluble or membrane components among mitochondria, providing critical support for vital functions such as oxidative phosphorylation, mitophagy, apoptosis, cell proliferation, and migration. Deletion of MFN1/2 or OPA1 in mice  is lethal^4^ and mutations in MFN1/2 and OPA1 in humans are linked to nervous system impairments like neuropathy^5^ and dominant optic atrophy^6,7^. Some stressors alter either the amount or the post-translational modification of fusion proteins to perturb the fission/fusion balance, leading to either hyperfusion or fragmentation of mitochondria, which changes help to adjust mitochondrial functions.

Mitochondrial membrane integrity is needed for cell survival since its loss leads to the release of mitochondrial intermembrane space (IMS) content, which compromises energy metabolism and activates death pathways. For instance, mitochondrial Ca^2+^ overload leads to permeability transition pore (PTP) formation in the inner mitochondrial membrane (IMM) with the ensuing damage of the outer mitochondrial membrane (OMM) leading to cell death^8^. Alternatively, in mitochondrial apoptosis, oligomerization of two pro-apoptotic Bcl-2 family proteins, Bak and Bax results in selective permeabilization of the OMM releasing IMS components like cytochrome c (cyto c), Smac/Diablo to the cytosol activating caspases and other executioner enzymes. Normally, Bak and Bax are neutralized by anti-apoptotic members of the Bcl-2 family like Bcl-2, Bcl-xL, and Mcl-1. However, many stressors target pro-apoptotic members of this family like Bid, Bim, Noxa, or PUMA to activate Bak/Bax directly or indirectly^9^. For instance, TNFα or Fas activates caspase-8 to truncate Bid, a pro-apoptotic protein to tBid that induces oligomerization of the OMM resident Bak or enhances translocation of the cytoplasmic Bax to the OMM where it also undergoes oligomerization to execute OMM permeabilization (OMMP)^10^. Death of a cell terminates its individual life, but may support survival of the whole organism undergoing stress^10^.

Excessive ethanol (EtOH) consumption causes tissue damage with 2.5 million deaths/year globally^11^. Alcoholic diseases are primarily attributed to the toxic metabolites of EtOH but more organs are affected than the ones metabolizing EtOH. EtOH metabolites are mainly produced in the liver by two enzymes; alcohol dehydrogenase (ADH) mainly in cytosol and Cytochrome P450 E2 (CYP2E1) in microsomes, producing acetaldehyde. Further, aldehyde dehydrogenase in mitochondria turns acetaldehyde into acetate and then Acetyl-CoA, utilized in mitochondrial metabolism^12^. Acutely EtOH also targets proteins and lipids in membrane and reduces membrane integrity, whereas chronic EtOH (chrEtOH) stiffens the membrane possibly by recruiting cholesterol^13^. Reactive oxygen species (ROS), a byproduct of EtOH metabolism, can denature proteins, break and therefore potentiate DNA to mutate, oxidize lipids, and generate products such as malonaldehyde and 4-hydroxynonenal^14^. These adducts and acetaldehyde, the instant product of EtOH oxidation, can further interact with biomolecules and modulate their function. Shifting the oxidative state of the mitochondrial matrix may compromise oxidative phosphorylation.

Mitochondria are a primary target for EtOH toxicity because they oxidize the highly reactive acetaldehyde and generate ROS^15^. We have shown suppressed mitochondrial fusion in several tissues of EtOH-fed rats^16,17^. As various organs differ in handling EtOH it is important to study the mitochondrial effects in both metabolizing and non-metabolizing conditions. To test whether the metabolites of EtOH were involved, we have studied here mitochondrial dynamics in HepG2 cells, which are available both with and without EtOH-metabolizing enzymes. Furthermore, we and others have shown that EtOH exposure reduces mitochondrial capacity to retain Ca^2+^ and promotes the Ca^2+^-induced PTP formation^18–20^ and subsequent membrane potential (ΔΨ_m_) dissipation^19,20^. We here investigated the effect of EtOH stress on the Bid/Bak pathway in the HepG2-derived cells.

## Results

### ChrEtOH-induced suppression of mitochondrial fusion dynamics depends on ADH and CYP2E1

We have shown suppressed mitochondrial dynamics in hepatocytes isolated from EtOH-fed rats^17^. To understand whether this phenomenon is dependent on EtOH metabolism, we used VL-17A, a hepatic cell line derived from HepG2 cells^17^, stably transfected to express ADH and CYP2E1 with high and low affinity to bind and convert EtOH to acetaldehyde (Figure S1). To compare VL-17A with a control, two options were available, the parental HepG2 cells, deficient in ADH and CYP2E1 activity^21,22^ and VI-7 (that carries the empty version of 1 (Zeocin-resistance) of the 2 vectors used to create VL-17A) that is ADH negative^23^. We confirmed the lack of the activity of both ADH and CYP2E1 in VI-7 cells (Figure S1A–D).

We tested both HepG2 and VI-7 cells for mitochondrial morphology and dynamics using mitochondrial matrix-targeted DsRed (mtDsRed) and photo-activatable green fluorescent protein (mtPA-GFP) as described before^24,25^. Based on the fluorescent protein distribution, both HepG2 and VI-7 cells show similar mitochondrial morphology (Figure S2A). After photoactivation, the overall level of mitochondrial dynamic activity, which includes fusion–fission and motility, was assessed by measuring the spreading of the mtPA-GFP from the photo-activated regions (Figure S2B). Because both VI-7 and HepG2 showed almost identical fluorescence spreading, in the rest of the studies of mitochondrial dynamics, we picked only one control, HepG2, to be compared with VL-17A cells.

**Fig. 1:**
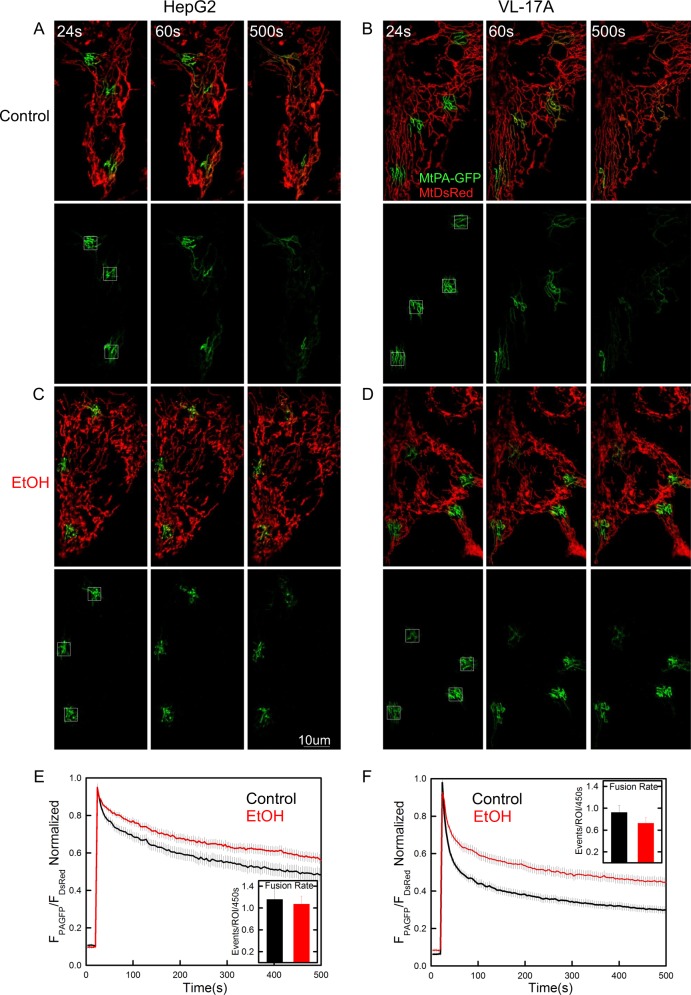
ChrEtOH perturbs mitochondrial morphology and dynamics significantly only in VL-17A cells. Cells were transfected with mitochondrial matrix-targeted DsRed (mtDsRed) and mtPA-GFP. Mitochondrial morphology and GFP spreading after photoactivation in designated areas are shown for a typical control HepG2 **a** and VL-17A cells **b** or the cells chronically treated with EtOH **c**, **d**. In the lower panels, only the green channel is shown. **e**, **f** Spreading rate of GFP after photoactivation has been quantified and presented as the normalized ratio of mtPA-GFP to mitoDsRed in the photo-bleached regions. **e**, **f** fusion rates were presented as the mean number of fusion events that calculated manually in the region of interests (ROI) for 450 s. **e** *n* for control and EtOH is 27 and 29; **f** *n* for both HepG2 and EtOH is 37) (*n* = 4)

VL-17A mitochondria were more elongated than HepG2 mitochondria. Chronic exposure to EtOH did not significantly alter mitochondrial morphology in HepG2 cells (Fig. 1c vs a) but shortened and fragmented mitochondria in VL-17A cells (Fig. 1d vs b). To specifically assess fusion state, the initial phase of the mtPA-GFP spreading was inspected because this represents the diffusion of mtPA-GFP among the mitochondria with continuous matrix. The spreading appeared slightly slower in the EtOH-treated HepG2 and markedly suppressed in the EtOH-treated VL-17A cells, indicating suppression of dynamics (Fig. 1e, f). To quantitatively compare the EtOH effect on both network continuity and fusion activity in the two cell lines, mtPA-GFP spreading values were compared at 156 s. Two-way analysis of variance (ANOVA) confirmed that: (1) untreated HepG2 shows slower spreading than untreated VL-17A (*p* < 0.001); (2) the effect of EtOH was significant only in VL-17A (HepG2: *p* = 0.09, VL-17A: *p* < 0.001); and (3) the effect of EtOH was significantly larger in VL-17A than in HepG2 cells (*p* < 0.001, Figure S2C black bars), indicating that the effect of chrEtOH depends on the presence of EtOH-metabolizing enzymes. To further assess mitochondrial fusion, the individual events were manually counted^24,25^. This revealed similar fusion activity in both HepG2 and VL-17A cells, and upon chrEtOH treatment, a decrease appeared in VL-17A cells but it was not significant (Fig. 1e, f).

### Application of EtOH metabolism-altering agents restores mitochondrial dynamics

**Fig. 2:**
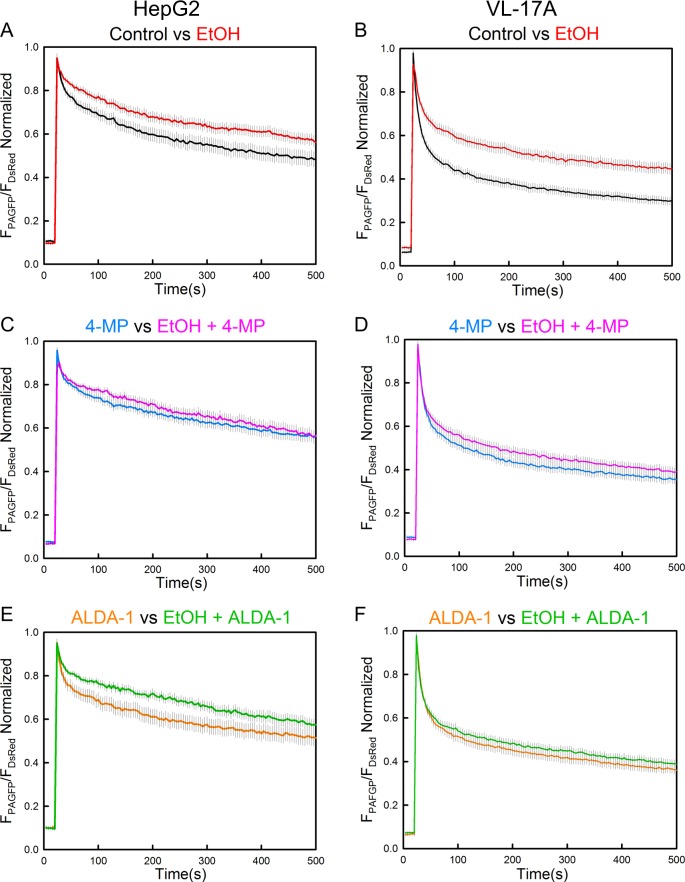
Drugs altering EtOH metabolism affect the response of mitochondrial dynamics to chrEtOH only in VL-17A cells. **a**, **b** GFP spreading in both EtOH and mock-pretreated HepG2 and VL-17A cells transfected with mtDSRed/mtPA-GFP (reproduced from Fig. 1e, f). **c**, **d** similar to **a**, **b** except that 5 mM 4-MP has been added to the medium simultaneous with EtOH (**c** *n* for 4-MP and EtOH+4-MP is 29 and 28; **d** *n* for 4-MP and EtOH+4-MP is 37). **e**, **f** similar to **a**, **b** except that 10 μM ALDA-1 has been added to the medium simultaneously with EtOH **e** *n* for ALDA-1 and EtOH+ALDA-1 is 27 and 28; **f** *n* for ALDA-1 and EtOH+ALDA-1 is 37) (*n* = 4)

To validate that the effect chrEtOH on mitochondrial dynamics was promoted by its metabolism, both HepG2 and VL-17A cells were treated either with 4-methylpyrazole (4-MP), an ADH enzyme inhibitor or ALDA-1, an agonist for aldehyde dehydrogenase 2 (ALDH2) simultaneous with chrEtOH treatment. In the presence of 4-MP, the EtOH effect was unaltered in HepG2 cells (Fig. 2c vs a, S2C), but was attenuated in VL-17A cells (Fig. 2d vs b, S2C). Similarly, treatment of the cells with ALDA-1 did not cause a change in HepG2 cells (Fig. 2e, S2C) but prevented the EtOH effect in VL-17A cells (Fig. 2f, S2C). Thus, any of the 2 agents interfering with EtOH metabolism showed rescue of mitochondrial dynamics in EtOH-treated VL-17A cells. Collectively, these results indicate that suppression of mitochondrial dynamics by EtOH is directly dependent on the activity of ADH and CYP2E1 enzymes and probably on acetaldehyde/ROS as the major EtOH metabolite/byproduct. Notably, both 4-MP and ALDA-1 suppressed fluorescent protein spreading by borderline significance in non-treated VL-17A cells (*p* = 0.043 and *p* = 0.05), suggesting that the basal level enzyme activity of ADH and CYP2E1 (Figure S1) and the pathway targeted by the drugs has some stimulatory effect on mitochondrial dynamics.

### EtOH treatment enhances ROS in mitochondria in VL-17A cells to reduce mitochondria dynamics

**Fig. 3:**
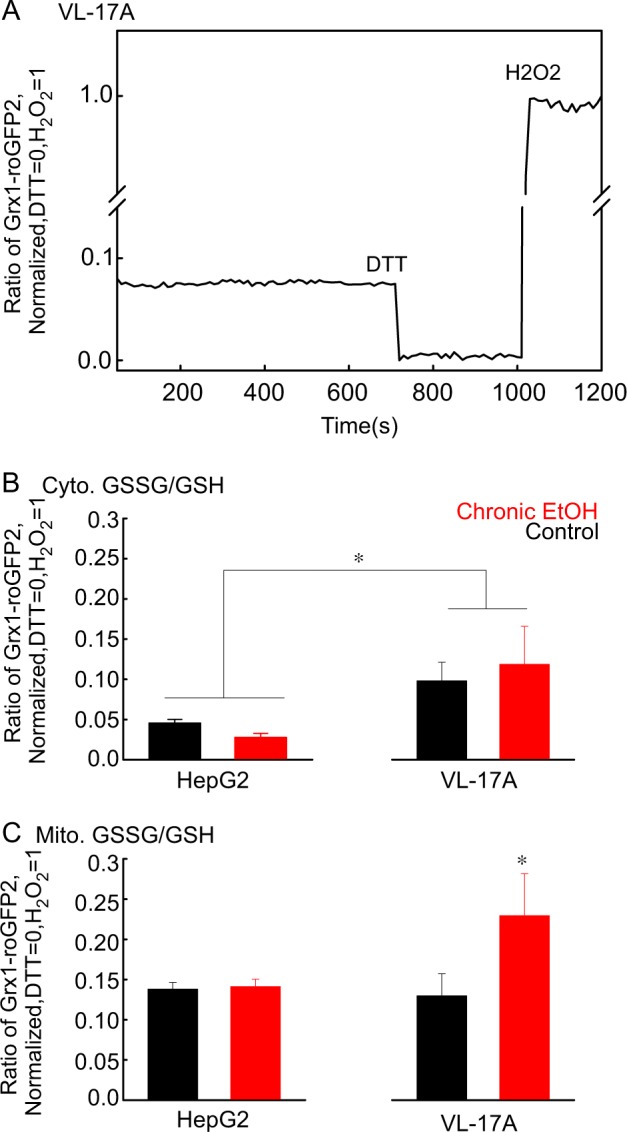
ChrEtOH exposure induces ROS increase in the mitochondria of VL-17A cells but not in HepG2 cells. **a** A representative time series recorded for oxidized glutathione (GSSG) to reduced glutathione (GSH) ratio using cytosolic Grx1-roGFP2. The trace was normalized to DTT response as minimum and H_2_O_2_ as maximum (zero and one). **b** Mean±SE of peak fluorescence ratio of cytosolic Grx1-roGFP2 values obtained from normalized individual traces is shown. *N* was 62 and 59 for untreated and chronically treated HepG2 cells and 13 and 20 for untreated and chronically treated VL-17A. **c** The same as B except that the probe is localized in mitochondria. *N* was 74 and 58 for untreated and chronically treated HepG2 cells and was 20 and 24 for untreated and chronically treated VL-17A (*n* = 4, *: *p* < 0.05)

**Fig. 4:**
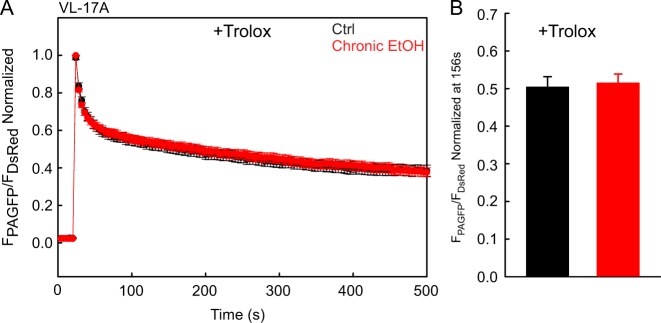
Trolox abolishes the effect of chrEtOH on mitochondrial dynamics in VL-17A cells. **a** Mean traces of time lapse of mtPA-GFP spreading in both EtOH and mock-exposed VL-17A cells, which were treated with 50 μM Trolox. **b** Mean value of mtPA-GFP spreading at 156 s. *N* was 34 and 38 for EtOH-treated and untreated cells (*n* = 3)

To determine whether ROS levels are differently affected by prolonged EtOH exposure in HepG2 and VL-17A, and to evaluate the subcellular localization of ROS, cells were transfected with a genetically encoded probe, Grx1-roGFP2, that measures the ratio of oxidized glutathione (GSSG) to reduced glutathione (GSH), targeted either to the cytoplasm or mitochondrial matrix. The fluorescence ratio of Grx1-roGFP2 specifically measures GSSG/GSH, reflecting ROS levels. As references, dithiothreitol (DTT) was used to determine the fully reduced and H_2_O_2_ to obtain the maximally oxidized states. A representative graph is shown in Fig. 3a and the baseline fluorescence ratios normalized to the range between DTT and H_2_O_2_ are shown for the cytoplasm in Fig. 3b and for the mitochondrial matrix in Fig. 3c. The cytoplasmic Grx1-roGFP2 signal indicated a more oxidizing environment in the VL-17A than in the HepG2 cells (Fig. 3b). Neither chronic (Fig. 3b) nor acute EtOH (Figure S2D) changed the baseline level of cytoplasmic ROS in HepG2 and VL-17A cells. The mitochondrial Grx1-roGFP2 signal was similar in HepG2 and VL-17A cells in the absence of EtOH, and was unaffected by acute EtOH addition (Figure S2E). However, chrEtOH treatment created a more oxidizing environment in the mitochondrial matrix of VL-17A cells compared with HepG2 cells (Fig. 3c). These results suggest that EtOH metabolism to acetate is associated with ROS production that causes an oxidative shift in the mitochondrial matrix, whereas in the absence of ADH and CYP2E1, ROS generation is undetectable. To test whether the ROS are relevant in the attenuation of mitochondrial fusion, a vitamin E-derived ROS scavenger, Trolox, was used. Trolox rescued the EtOH-induced suppression in VL-17A cells (Fig. 4), indicating that ROS is responsible for suppression of mitochondrial dynamics by prolonged EtOH in VL-17A cells.

### ChrEtOH does not alter ΔΨ_m_ and Ca^2+^ uptake or the abundance of fusion proteins

**Fig. 5:**
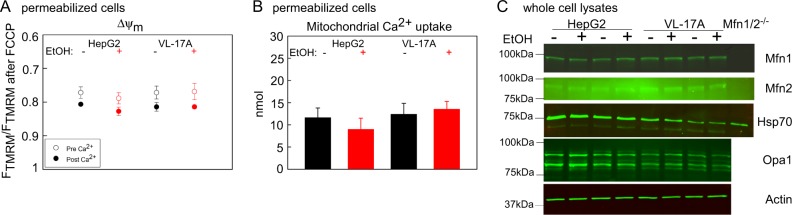
ΔΨm, Ca^2+^ handling, and the abundance of the mitochondrial fusion proteins are unchanged in chronically EtOH-treated VL-17A and HepG2 cells. **a** ΔΨ_m_ before and after the addition of Ca^2+^ were presented as the fluorescent intensity of TMRM normalized to the TMRM fluorescent intensity obtained after complete depolarization by FCCP (5 μM) in permeabilized cell suspensions (*n* = 3). **b** Mitochondrial Ca^2+^ uptake was measured using Fura2FF and the data presented as the nmol Ca^2+^ (*n* = 3). **c** Western blot for Mfn1/2 and Opa1 in the cell lysates of VL-17A and HepG2 cells, which were chronically treated with EtOH. Actin was used as loading control (*n* = 3)

Because both mitochondrial fusion and motility are affected by ΔΨ_m_ and Ca^2+^^25,26^, we hypothesized that these factors might be involved in the suppression of mitochondrial dynamics. First, we compared ΔΨ_m_ values, before and after mitochondrial Ca^2+^ uptake in VL-17A and HepG2 cells. Both HepG2 and VL-17A cells showed similar ΔΨ_m_ before and after Ca^2+^ addition, and no change in ΔΨ_m_ occurred when they were exposed to EtOH (Fig. 5a). Mitochondrial Ca^2+^ uptake was also unaffected by chrEtOH (Fig. 5b).

We also investigated if EtOH metabolites or generated ROS might affect the levels of the fusion proteins. Western blots (Fig. 5c) and the quantification of relevant bands (Figure S3) showed no decrease in the abundance of Mfn1/2, Opa1 in HepG2 and VL-17A cell lines exposed chronically to EtOH. Thus, EtOH-induced ROS suppresses mitochondrial fusion dynamics independent of ΔΨ_m_, Ca^2+^ handling and the abundance of fusion proteins.

We have already shown that ROS can sensitize cells to tBid-induced OMMP^27^. Based on the above results, we were wondering whether EtOH also sensitizes the cells to tBid primarily through its metabolism. We first set out to study the effect of EtOH on HepG2 cells that we considered as a “negative control” because of the lack of ADH and CYP2E1 enzyme activity^21,22^.

### EtOH sensitizes HepG2 and heart-derived H9c2 cells to tBid-induced OMMP and cell death

**Fig. 6:**
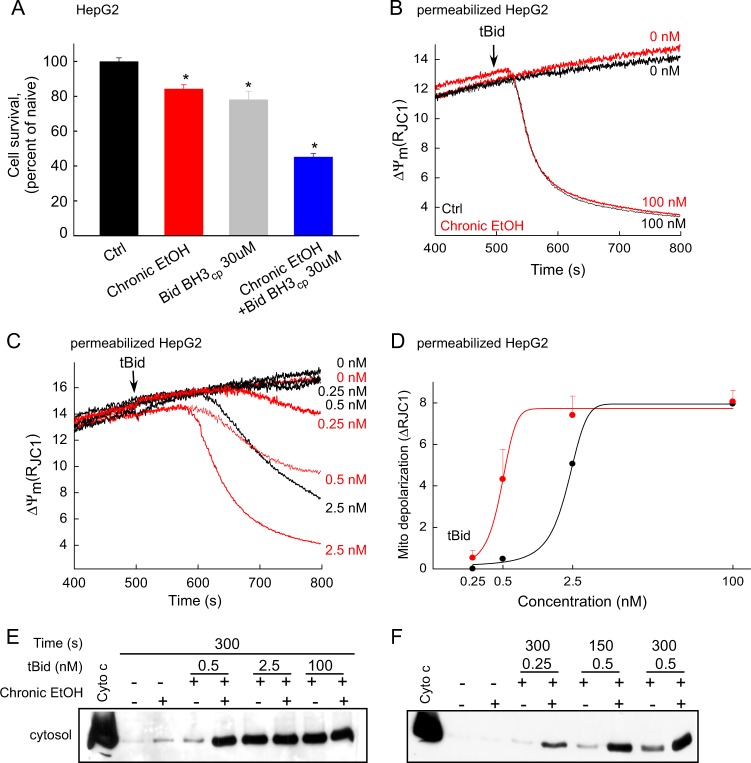
EtOH sensitizes HepG2 cells to tBid-induced OMMP. **a** Cell viability was tested for 100 mM EtOH and/or 30μM Bid BH3_cp_-treated intact HepG2 cells (*n* = 4, *p < 0.05). **b** Time-course recording of ΔΨ_m_ in permeabilized HepG2 cell suspension using fluorometer. 0, 100 nM tBid was added at 500 s. Red traces represent EtOH-treated cells and black traces non-treated ones. **c** Similar to **b** except lower concentrations of tBid (0, 2.5, 0.5, and 0.25 nM) were used (*n* = 3). **d** Dose–response relationship between tBid concentration and ΔΨ_m_ dissipation in HepG2 permeabilized cells. **e** Immunoblot of cyto c in the cytosolic fraction of the permeabilized cells that were separated quickly from pellet fraction right after 300 s treatment with tBid or solvent. Cyto c was loaded as a positive control. **f** Similar to **e** except the treatment times were either 300 s or 150 s

To study the direct effect of EtOH on tBid-induced cell death, HepG2 cells were pretreated with either 0 or 100 mM EtOH for 72 h. Zero or 30 μM cell permeable Bid BH3 peptide (Bid BH3cp)^28^ was added to each in the last 2 h before cell viability was tested. Viability was reduced by ~ 20% in cells exposed to either EtOH or Bid BH3cp alone, and by ~ 60% when Bid BH3cp was added to the cells that were pretreated with EtOH, indicating that unexpectedly, EtOH independent of its metabolism, sensitizes the cells to tBid-induced cell death (Fig. 6a).

To study the involvement of mitochondria directly, tBid was added to EtOH-pretreated and untreated plasma membrane-permeabilized HepG2 cells in suspension. tBid-induced OMMP was measured as cytochrome c (cyto c) redistribution from mitochondria to cytosol and ΔΨ_m_ collapse measured in the presence of oligomycin. Addition of a high concentration of tBid, 100 nM, resulted in a complete ΔΨ_m_ loss with half-maximal response ~ 50 s in both EtOH-pretreated and untreated cells (Fig. 6b). Addition of 2.5 nM and less tBid caused delayed ΔΨ_m_ dissipation with a slower rate (Fig. 6c). Under this condition, ΔΨ_m_ dissipates faster in EtOH-treated cells compared with naive. The difference between EtOH-treated and untreated cells was more striking at lower tBid concentrations (i.e., 0.5 nM and 0.25 nM). In fact, this concentration of tBid caused mitochondrial depolarization only in EtOH-pretreated cells (Fig. 6c). Figure 6d shows the tBid dose–response relationship for both untreated cells and EtOH-pretreated ones and reveals a leftward shift in the EtOH condition. To study the distribution of cyto c, cytosolic and membrane fractions were separated and the purity of each fraction was assessed by α-Tubulin, mitochondrial HSP70, and Prohibitin as cytosolic or mitochondrial marker (Figure S4A). Western blot analysis of cytosolic fractions confirmed that addition of high tBid for 5 min results in similar cyto c release in both EtOH and untreated cells (Fig. 6e). Because the release of cyto c is an earlier event than the ΔΨ_m_ collapse, whereas EtOH-treated cells showed higher sensitivity to ΔΨ_m_ collapse after addition of 2.5 nM tBid for 5 min, both EtOH-exposed and non-exposed cells showed similar cyto c release (Fig. 6e). With lower concentrations of tBid (0.25 and 0.5 nM) in short/long time points, a larger fraction of cyto c was released in the EtOH-pretreated samples (Fig. 6). To assess whether EtOH favors the release of multiple IMS pro-apoptotic proteins, Smac/Diablo release was also tested in the tBid-treated samples using western blot. High concentrations of tBid (2.5, 100 nM for 5 min) released Smac/Diablo similarly in both EtOH and mock-pre-exposed samples but in response to a submaximal concentration of tBid (0.5 nM) the EtOH-preexposed sample showed more release of Smac (Figure S4B). Collectively, these results show that EtOH sensitizes HepG2 cells to tBid-induced OMMP and release of multiple IMS proteins.

To test whether this phenomenon is cell line specific, we pretreated rat cardiac myoblasts, H9c2 cells, with EtOH for 72 h. H9c2 cells are more sensitive to tBid than HepG2 cells. As Figure S4C shows, a maximally effective dose of 2.5 nM tBid (as high concentration) caused ΔΨ_m_ dissipation with similar onset and kinetics in EtOH-pretreated or control cells with half-maximal response around 50 s. However, similar to HepG2 cells, a subthreshold concentration of tBid (0.032 nM) for the control caused ΔΨ_m_ loss in 40 or 80 mM EtOH-pretreated cells (Figure S4D). The velocity of depolarization depended on the EtOH concentration. Accordingly, low doses of tBid release cyto c only in EtOH-treated cells (Figure S4E). Thus, EtOH promotes tBid-induced OMMP in both HepG2 and a cell line derived from a tissue with little capacity to metabolize EtOH.

### EtOH sensitizes to tBid through Bcl-2 family proteins and independent of PTP opening

**Fig. 7:**
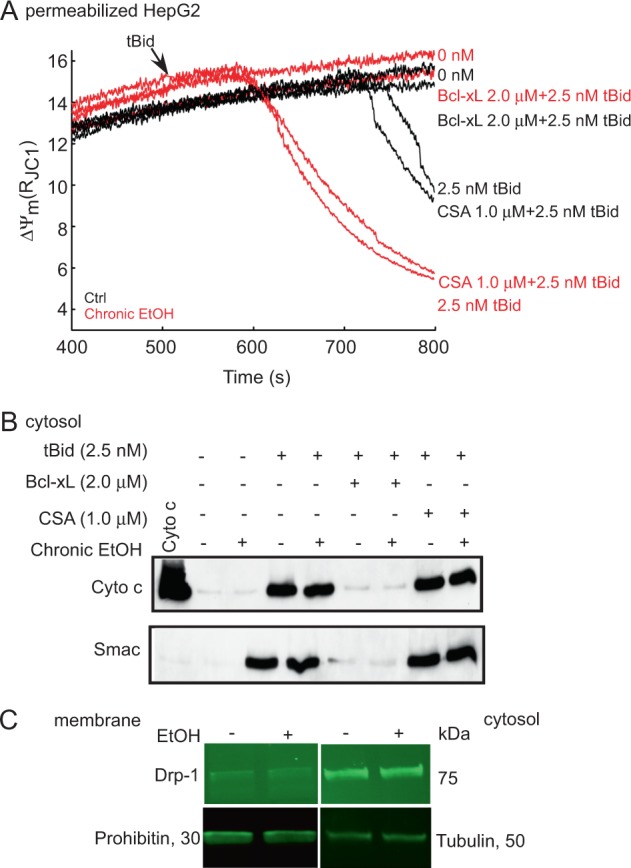
Bcl-xL desensitizes mitochondria to tBid-induced permeabilization independent of EtOH presence and CSA has no influence on EtOH-mediated sensitization to tBid. **a** Time-course recording of ΔΨ_m_ in the permeabilized HepG2 cells suspensions treated with 1 μM CSA or 2.5 nM Bcl-xL. tBid (0 or 2.5 nM) was added at 500 s. Arrow shows the tBid addition. Red lines represent 100 mM EtOH-pretreated, and black lines mock-treated cells (*n* = 3). **b** Western blot of cyto c and Smac/Diablo in the cytosolic fraction of the samples that were treated with 1 μM CSA, 2.5 nM Bcl-xL and tBid (0 or 2.5 nM) for 5 min. Cyto c was loaded as positive control. **c** Immunoblot of Drp1 in the membrane/cytosolic fraction of permeabilized HepG2 cells. Tubulin and Prohibitin were used as loading controls

To test whether the EtOH-mediated sensitization effect requires PTP opening, we repeated the experiments shown in Fig. 6b both in the presence or absence of cyclosporine A (CSA), a PTP inhibitor (Fig. 7a). CSA failed to influence the onset or velocity of tBid-induced ΔΨ_m_ depolarization. To clarify if the effect of EtOH was mediated through Bcl-2 family proteins we used recombinant Bcl-xL^29^. Addition of anti-apoptotic Bcl-xL (2 μM) repressed tBid-induced ΔΨ_m_ loss in both the presence and absence of EtOH (Fig. 7a). Immunoblotting against cyto c and Smac showed that the tBid-induced cyto c/Smac relocation to the cytoplasm in both EtOH-exposed and non-exposed cells was unaffected by CSA treatment (Fig. 7b) and was blocked by Bcl-xL addition. Collectively, this shows that EtOH does not engage in PTP formation and likely facilitates pro-apoptotic Bcl-2 family proteins to respond to tBid.

### EtOH does not alter DRP1 expression or localization

DRP1^30,31^ and OPA1 proteins^32^ both are associated with the control of cyto c release. We have already shown that EtOH does not alter OPA1 level (Fig. 5c and S3) and we show in Fig. 7c that DRP1 was mainly in the cytosolic fraction of HepG2 cells and its abundance was unaffected by prolonged EtOH exposure.

### EtOH-dependent sensitization of tBid-induced OMMP is insensitive to ROS scavenging by Trolox

**Fig. 8:**
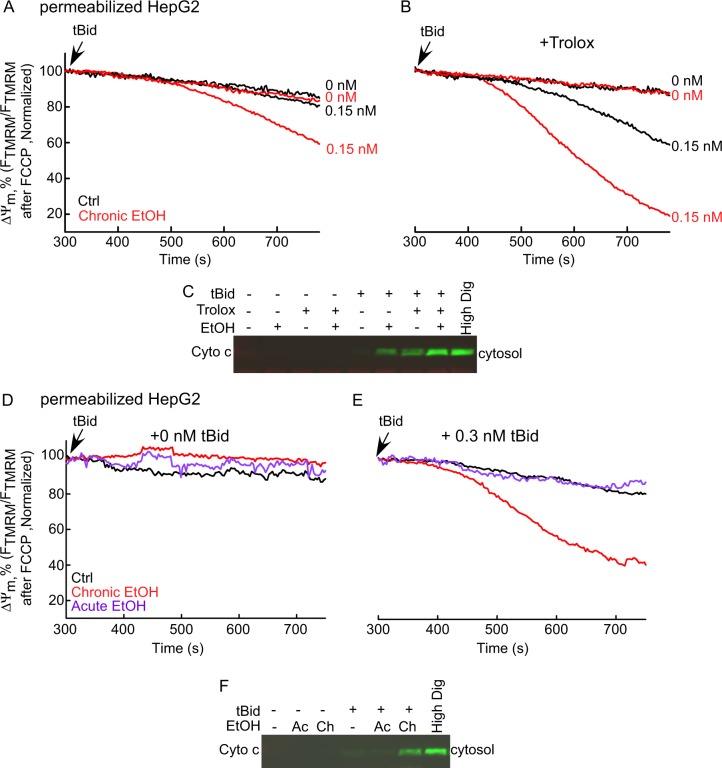
Trolox does not alter the EtOH-dependent sensitization of tBid-induced OMMP in HepG2 cells. Only chrEtOH sensitizes the cells to tBid. **a** Time-course graph of ΔΨ_m_ in 0 and 100mM EtOH-exposed permeabilized HepG2 cells. tBid (0 and 0.15 nM) was added at 300 s. Similar to Fig. 6b, at the end of each experiment, FCCP was added. The data are presented as the percentage of the initial ΔΨ_m_. Arrow shows the tBid addition. **b** Similar as **a**, except that the cells have been treated with 50 μM Trolox for 72 h before the experiment and was refreshed every 24h (*n* = 3). **c** Western blot of cyto c in the cytosolic fraction of the HepG2 permeabilized samples, which were separated from the pellet at the end of each run. High concentration (600 μg/ml) of Digitonin was used to release all the cyto c in the mitochondria as positive control. **d**, **e** Mean traces of time-course recording of ΔΨ_m_ in permeabilized HepG2 cells suspension in cells chronically or acutely exposed to 100 mM ethanol (*n* = 3). tBid concentration was either zero **d** or 0.3 nM **e**. At the end of each experiment, FCCP (5 μM) was added to depolarize the remaining ΔΨ_m_. The data are presented as the percentage of the initial ΔΨ_m_. Arrow shows the tBid addition. Black, red, and purple traces are control, chronically and acutely EtOH-treated in order. **f** Western blot of cyto c

As shown in Fig. 3, there is no change in the ratio of GSSG to GSH in EtOH-treated HepG2 cells suggesting that global ROS was not increased; however, this did not exclude a local ROS increase close to the mitochondria. To test the involvement of ROS, cells were pretreated with/without 50 μM Trolox simultaneously with EtOH. Treatment with Trolox failed to suppress EtOH’s effect on tBid-induced cyto c release and mitochondrial depolarization (Fig. 8a–c). In fact, Trolox exerted some sensitization to tBid-induced OMMP both in the absence and presence of EtOH treatment. This set of data further supports that the influence of EtOH on OMMP is not mediated through ROS.

### Prolonged exposure to EtOH is required to sensitize tBid-induced OMMP

Next, we studied whether EtOH acts instantaneously or long-term EtOH exposure was needed. HepG2 cells were exposed to both chrEtOH (72 h) and acute EtOH and ΔΨ_m_ and cyto c release were recorded upon tBid treatment. Although chrEtOH sensitized cells to tBid, acute exposure failed to show any effect on tBid-induced OMMP (Fig. 8d–f).

### EtOH’s facilitation of the tBid pathway is independent of its metabolism

**Fig. 9:**
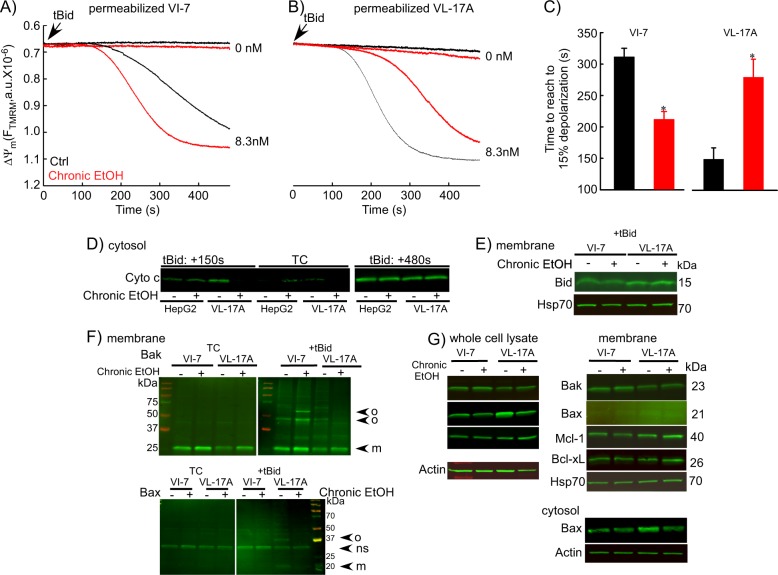
EtOH sensitizes HepG2 via increased Bak oligomerization but fails to sensitize VL-17A cells to tBid-induced OMMP. **a**, **b** Representative time-course recording of ΔΨ_m_ in permeabilized VI-7 **a** and VL-17A **b** cell suspension. Arrows show the addition of zero or 8.3 nM tBid. **c** Bar charts show the average of the time to reach to 15% of the depolarization upon treating the VI-7 and VL-17A cells with 8.3 nM tBid as described in Fig. 8a, b. (*: *p* < 0.05, *n* = 4). **d** Western blot using anti-cyto c antibody for the cytosolic fraction of the EtOH-exposed or non-exposed VI-7 and VL-17A cells. Permeabilized cells were treated with tBid for 150 s (left panel) or 450 s (right panel) and as described in Fig. 6e. TCs are the samples that were not treated with tBid. **e** Western blot using anti-Bid in the membrane fraction of EtOH-exposed and non-exposed VI-7 and VL-17A cells that were treated with 8.3 nM tBid. Hsp70 was used as loading control. **f** Western blot using anti-Bak (upper panels) and anti-Bax (lower panels) in the membrane fraction lysates of ethanol exposed and non-exposed VI-7 and VL-17A cells, which were treated with either zero (left panels) or 8.3 nM tBid (right panel). Membrane fractions were separated from cytosol and were treated with BMH (poly-linker) as described in Materials and methods. (O: oligomeric, ns: non-specific band, m: monomeric band) **g** Western blot of whole cell and membrane fraction lysates or cytosol fraction of the ethanol exposed and non-exposed VI-7 and VL-17A cells using anti-Bak, -Bax, -Mcl-1, -Bcl-xL. Actin or Hsp70 was used as loading control

To test whether metabolism of EtOH enhances the sensitization to tBid we studied VI-7 and VL-17A cells. As shown before for HepG2 (Figs. 6–8), EtOH-pretreatment of VI-7 resulted in higher tBid-induced depolarization velocity and cyto c release (Fig. 9a, c, d). Untreated VL-17A were more sensitive to tBid than VI-7, but unexpectedly, EtOH failed to sensitize tBid-induced OMMP (Fig. 9b–d) and actually, in this condition EtOH-exposed VL-17A showed slower depolarization and less cyto c release (Fig. 9d). When the tBid treatment was more prolonged (480 s), both HepG2 and VL-17A showed a similar level of cyto c in the presence or absence of EtOH.

To further explore this, we studied whether EtOH promoted the interaction of tBid with the OMM. Western blotting against Bid (Fig. 9e), showed similar amounts of the added tBid in the membrane fraction of EtOH-exposed and non-exposed cells, indicating that tBid interaction with the membrane was unchanged by EtOH. Using a cross-linker, we found that tBid-induced OMMP is mainly the result of Bak and not Bax oligomerization in VI-7 and that EtOH exposure increases Bak oligomerization in this cell type (Fig. 9f). By contrast, EtOH-exposed VL-17A showed lesser Bak oligomerization compared with the controls. In addition, only in VL-17A, tBid induced Bax oligomerization, which was suppressed in EtOH-exposed VL-17A (Fig. 9f, lower).

Finally, we asked if EtOH treatment would alter the amount of Bcl-2 family proteins required to sensitize cells to tBid. Most of the relevant Bcl-2 family members in the tBid-OMMP pathway (Bak, Bcl-xL, and Mcl-1) did not show any differences in the protein levels (Fig. 9g). One exception is that Bax levels were relatively high in untreated VL-17A cells, and that was reduced after EtOH treatment (Fig. 9g, S5A). Another mechanism for the sensitizing effect of chrEtOH could be an elevation of endogenous tBid or Bim, which might occupy the anti-apoptotic Bcl-2 family proteins. However, quantification of BimEL, BimL, BimS, and tBid showed no change upon EtOH treatment (Figure S5B).

Collectively, the results on HepG2/VI-7 vs VL-17A indicate that EtOH sensitizes tBid-induced OMMP independent of EtOH metabolism and changes in levels of Bcl-2 family proteins. The effect of EtOH is downstream of tBid association to the OMM, and likely occurs at the level of Bak oligomerization. Furthermore, the results also revealed a relatively high tBid sensitivity in VL-17A cells possibly owing to higher Bax abundance and tBid-induced Bax oligomerization. Unexpectedly, only in the EtOH-metabolizing condition, Bax was downregulated, and both Bak and Bax oligomerization are attenuated.

## Discussion

We studied the effect of EtOH-induced stress on mitochondrial fusion and mitochondrial apoptosis in VL-17A cells that serve as a simplified model of hepatocytes, and HepG2/VI-7 cells lacking the EtOH-metabolizing enzymes. Previously, the effect of chrEtOH on intracellular signaling and dynamics was investigated mainly in EtOH-fed animal models, in which EtOH effects might be influenced by several other factors or in cell lines that do not metabolize EtOH. The present paradigm allows direct application of EtOH to cell types, which are identical except the difference in EtOH-metabolizing enzymes.

We showed that EtOH metabolism in VL-17A leads to ROS elevation in the mitochondrial compartment but in HepG2, EtOH fails to increase mitochondrial ROS. As the ROS elevation is confined to the mitochondria, the reason of ROS elevation can be an increase in the respiratory chain activity, change in the redox state (change in NAD/NADH ratio) and reduction of antioxidants-like superoxide dismutase^33^ and elevation of Glutamate dehydrogenase activity^34^. Using a ROS scavenger, we linked EtOH-induced suppression of mitochondrial dynamics to ROS generation.

We documented that mitochondrial fusion is suppressed in VL-17A cells upon chrEtOH similar to hepatocytes^17^ without a change in fusion protein levels. Therefore, we reason that a change in the lipid membrane or translated fusion proteins or mitochondrial positioning affected fusion activity. ROS elevation might result in lipid peroxidation or addition of residues (like HNE) to lipids followed by alteration in the physiochemical properties of the membranes. Evidence has been presented that EtOH causes modification of cardiolipin in the liver^35,36^. Cardiolipin has also been described as a factor required for both OPA1-mediated IMM fusion^37^ and MFN-mediated OMM fusion^38^. In addition, cardiolipin interacts with DRP1 to facilitate fission^39^, and is needed for crista formation^40^. Thus, the EtOH-induced fusion suppression might involve modification of cardiolipin but because of the wide range of cardiolipin-dependent processes, this possibility is difficult to directly test.

Whereas reduced mitochondrial fusion in VL-17A was independent of fusion proteins levels, we showed that in skeletal muscle fibers^16^ extracted from chrEtOH-fed rats, mitochondrial fusion was suppressed through a decrease in MFN1 protein level. Both skeletal muscle and brain have little capacity to metabolize EtOH. Dysregulation of ΔΨ_m_ or Ca^2+^ transport could also cause a decrease in mitochondrial fusion. In hepatocytes isolated from EtOH-fed animals, Gaspers et al.^41^ reported higher levels and uptake of mitochondrial Ca^2+^, attributed to higher expression of VDAC (voltage-dependent anion channel) and MCU (mitochondrial Ca^2+^ uniporter) proteins. However, in VL-17A or HepG2 cells, EtOH treatment did not change mitochondrial Ca^2+^ uptake or ΔΨ_m_ excluding the role of these factors in fusion inhibition.

Toxicity depends on the concentration and exposure time of EtOH. High doses of EtOH lead to mitochondrial Ca^2+^ overload, ΔΨ_m_ dissipation, release of cyto c, and cell death^42^. In this study, 80–100 mM EtOH for 48–72 h caused only a small increase in cell death in HepG2 cells. No sign of PTP formation or cyto c release were detected, as in^43,44^. However, we showed that EtOH sensitizes HepG2 cells to tBid-induced OMMP and cell death. Pastorino et al.^45^ have already shown that EtOH can potentiate mitochondrial membrane permeabilization by TNF-α, upstream of tBid in HepG2 cells, however; they showed that this effect is independent of caspase-8, which is needed for activation of Bid to tBid. Thus, EtOH seems to sensitize cells to TNF-α-dependent pathways in multiple ways. Along this line, it is likely that EtOH would affect mitochondrial cell death induced by drugs with many cellular targets, including the Bak/Bax pathways like staurosporine and etoposide but this effect would not be specific to the tBid-Bak pathway.

EtOH can directly target cellular membranes and causes disordering, disrupting, or altering of the physical shape of the membrane and therefore changing the ultrastructure of mitochondria. However, as short-term EtOH exposure does not promote tBid-induced OMMP in HepG2 cells, acute effects on the membranes are not the reason for EtOH-induced sensitivity to tBid. As cyto c is located in the cristae, it is possible that EtOH-induced morphology impairs the interaction between cyto c and its binding partner in IMM. In animals, chrEtOH stiffens the membranes by increasing cholesterol and saturated lipids^13^. In mitochondrial membranes, cardiolipin confers resistance to EtOH^36^. The potential alterations in lipids may be relevant for the tBid-induced OMMP. On the other hand, EtOH might interact with proteins in the membrane and change their conformation^46^. Indeed, in another paradigm, EtOH has been described to associate with phospholipase C and interfere with PLC/D mediated signal transduction^47,48^.

EtOH has been described to alter Bcl-2 family proteins at the level of mRNA or protein^42,49–52^. However, we only found Bax levels affected in VL-17A. Furthermore, EtOH has been proposed to affect the post-translational modification of proteins in non-metabolizing cells^53,54^ that might also contribute to tBid-induced OMMP. Recently, Sariyer et al.^55^ showed that in neuronal cells chrEtOH exposure causes Mcl-1 missplicing and reduces the anti-apoptotic isoform of Mcl-1 but our data did not show this in HepG2 cells. The most relevant mechanistic finding here was that chrEtOH facilitated the tBid-induced Bak oligomerization that alone can explain the increased OMMP in HepG2 cells.

EtOH has been reported to enhance membrane permeability of mitochondria isolated from hepatocytes upon stimuli like high Ca^2+^, Bax protein, Ceramide etc^56^. Because EtOH-metabolizing VL-17A cells display elevated ROS and ROS were shown to sensitize the cells to tBid-induced cell death^27^, EtOH was anticipated to potentiate tBid/Bak-induced death in VL-17A cells. This idea was also supported by data on VL-17A cells that upon exposure to EtOH, oxidative stress, caspase 3 activation, and cell death were observed^44,57^. Surprisingly, we found that chrEtOH exposure desensitized VL-17A to tBid-induced OMMP. Bax, a target of tBid is high in VL-17A cells but, Bax was reduced after chrEtOH like in hypoxia/reoxygenated brain^58^, providing a plausible explanation for inhibition of tBid-induced OMMP. It is worth noting that VL-17A treated with EtOH showed less mitochondrial connectivity and dynamics (Fig. 1) and in the same condition, it showed less Bax level and therefore less sensitivity to tBid-induced OMMP (Fig. 9). These two responses are not in conflict with each other as, e.g., mitochondria in Mfn1/2 KO MEFs are desensitized to tBid-induced OMMP^59^. Furthermore, Hoppins et al.^60^ showed that cytosolic Bax positively regulates mitochondrial fusion, which might explain that a decrease in both total Bax and elongated mitochondria appear together in EtOH-treated VL-17A cells. In addition, fragmentation is not necessarily less healthy condition, e.g., in the pro-survival phenomenon of autophagy mitochondrial fragmentation seems to be necessary^61^.

Our work described two different effects of chrEtOH-induced stress on mitochondria, which are oppositely dependent on EtOH metabolism. ChrEtOH by itself can exert some suppression of mitochondrial fusion but products of EtOH metabolism, acetaldehyde and ROS, exert a more profound inhibition. On the other hand, EtOH by itself sensitizes Bid-induced apoptosis independent of ROS and the products of EtOH metabolism seem to have an opposite effect. These observations might have medical significance in the injury of the various tissues in alcoholics. The metabolism-dependent effects have to be most prominent in the liver, whereas the ones caused by EtOH by itself should dominate in skeletal-or cardiac muscle. Although we got some clues to the targeting of MFN1 and BAK by EtOH, further efforts will be needed to decide if alteration of lipid profiles or post-translational modification of proteins plays a role in these mitochondrial stress responses.

## Materials and methods

### Chemicals

Cell permeable Bid BH3 peptide (Bid BH3_cp_) ([Arg](9)-Gly-Glu-Asp-Ile-Ile-Arg-Asn-Ile-Ala-Arg-His-Leu-Ala-Gln-Val-Gly-Asp-Ser-Met-Asp-Arg) was purchased from Selleckchem. tBid was produced as described^29^. Bismaleimidohexane (BMH) was from Pierce. G418 was from Enzo, Zeocin from Invitrogen. ALDA-1 from Tocris. Other chemicals except the specified ones were from Sigma.

### Cell lines, medium, and treatments

HepG2 cells (ATCC) were grown in minimum essential medium (ATCC) supplemented with 10% fetal bovine serum (FBS) (Gibco), 100 U/ml penicillin, 100 μg/ml streptomycin, and 2 mM Glutamine (Gibco) and kept in 37 °C and 5% CO2. Two modified HepG2 cell lines: (1) VL-17A cells expressing active murine alcoholic dehydrogenase class I (ADH) and human cytochrome P4502E1 (CYP2E1) similar to normal hepatocytes, and (2) VI-7 cells, that carry only one empty vector (Zeocin^+^) were from Dr. Dahn Clemens (Nebraska University, Omaha)^23,44,62^. VI-7 and VL-17A were cultured in the same medium as HepG2 with 400 μg/ml Zeocin for both (1) and (2) and with 400 μg/ml G418 antibiotics for (1). As neither HepG2 nor VI-7 show ADH and CYP2E1 activity and both cell lines show negligible EtOH metabolism activity^21,22^ (Figure S1), and similar mitochondrial dynamic activity (Figure S2) the presence of Zeocin in the culture medium does not seem to alter these activities and in the study of mitochondrial dynamics (Figs. 1–5) we could use HepG2 cells as the primary control. In the study of tBid sensitivity both HepG2 (Figs. 6–8) and VI-7 (Fig. 9) cells were used as control for VL-17A cells.

H9c2 cells (ATCC) were maintained as it was described before^20,27^. In acute treatments, 50–100 mM EtOH was added immediately before the experiment and in chronic treatments 50–100 mM EtOH was added for 48–72 h to the cells medium with every 12 h refreshments and every 24 h replacement. Plates were sealed to avoid EtOH evaporation. Trolox (50 μM), 4-MP (5 mM), and ALDA-1 (0.010 mM) treatments were performed at the same time as EtOH was administered.

### Transient expression

DNA transfection was performed using either XTremeGene9 (Roche) or Lipofectamine 3000 (Invitrogen) in accordance with the manufacturers’ protocols 24–48 h before imaging. The following plasmids were used: mtDsRed (Takara Bio Inc.) and mtPA-GFP^63^, which used the targeting sequence of cyto c oxidase subunit VIII. Grx1-roGFP2^64^, which is targeted to either cytosol or mitochondria using a signal sequence from Neurospora crassa ATP synthase protein 9.

### Live-cell microscopic imaging

Fluorescence spreading experiments were performed on a LSM780 microscope with a × 63 oil objective (Carl Zeiss), recording 512 × 512 pixel image pairs at 0.25 s^−1^. A Chameleon laser (760 nm, Coherent) was used for two-photon photoactivation of mtPA-GFP^25^. An argon laser source was used for imaging of mtPA-GFP at 488 nm and a DPSS laser at 568 nm for mtDsRed (Takara Bio). Image analysis was performed using Spectralyzer (MitoCare proprietary) software and/or Image-J (imagej.nih.gov).

ROS measurements were performed with an inverted epifluorescence microscope (Olympus) with a × 40 oil objective connected to a cooled CCD camera (PXL, Photometrics). Ratiometric imaging was performed at 490 nm and 415 nm, recording 512 × 512 or 256 × 256 pixel image pairs at 0.1 s^−1^. Calibration of the probe was performed by adding 2 mM DTT for minimal ratio value and 0.2 mM H_2_O_2_ for maximal ratio value. All the experiments were performed in extracellular matrix containing 0.25% bovine serum albumin at 37 °C.

### Bioluminescent viability assay

HepG2 cells were seeded in 24-well plate (24,000/well), treated with EtOH as it described in “Cell lines, medium, and EtOH treatment”. In the last 2 h, cells were treated with Bid BH3cp in the cell medium described above except that it was supplemented with 2% FBS. The cell viability measured using CellTiter-Glo Luminescent Cell Viability Assay (Promega).

### ΔΨ_m_ measurement, cyto c release assay, and Ca^2+^ recording in cell suspension

ΔΨ_m_ was recorded using fluorometer (DeltaRAM; Horiba, NewJersey, USA) with either TMRM (1.5 μM) or JC-1 (0.8 μM) as described^27,65,66^. Cells were harvested and washed with cold Na-Hepes-EGTA buffer containing 120 mM NaCl, 5 mM KCl, 1 mM KH_2_PO_4_, 0.2 mM MgCl_2_, and 20 mM Hepes-NaOH, pH 7.4. In 37 °C and under stirring condition, the same aliquots of cells (1.2–2.4 mg) were permeabilized using 30–40 μg/ml Digitonin in 1.5 ml intracellular medium buffer (ICM:120 mM KCl,10 mM NaCl,1 mM KH_2_PO_4_, 20 mM Hepes-Tris, pH 7.2) supplemented with 5 μg/ml protease inhibitors leupeptin, antipain, and pepstatin for 5 min. In all the experiments, 2 mM MgATP, 2 mM succinate, and 5 μg/ml oligomycin were present. Digitonin 600 μg/ml was used to release total cyto c from the mitochondria. Uncoupler, FCCP (5 μM) was applied at the end of each run to dissipate remaining ΔΨ_m_. JC-1 was used as the ratio of the aggregated dye intensity (570 nm excitation/595 nm emission) to monomeric dye (490 nm excitation/535 nm emission) and TMRM with 540 nm excitation and 580 nm emission. After each run samples were centrifuged for 5 min at 10,000×*g* and cytosolic and membrane fractions were separated. For Ca^2+^ measurements, 2 μM thapsigargin (Enzo) and 1.0 μM fura2FF (*Kd* = 4.5 μM, TEFLabs) were added to ICM, which was supplemented as explained above without oligomycin. Calibration for maximum and minimum fura2FF response was performed by adding 1 mM Ca^2+^ and 10 M EGTA/TRIS pH 8.5, respectively.

### Western blotting

Intact cells or membrane fraction (pelleted by centrifuging permeabilized cell suspensions at 10,000 × *g* for 5 min) were lysed in cold radioimmunoprecipitation assay buffer, (150 mM NaCl, 1.0% (vol/vol) Octylphenoxypolyethoxyethanol, 0.5 % sodium deoxycholate, 0.1 % sodium dodecyl sulphate, and 50 mM Tris (pH 8.0; Sigma)), supplemented with 1 μg/ml protein inhibitors (leupeptin, antipain, and pepstatin) and 1 mM phenylmethylsulfonyl fluoride. Lysed samples and cytosol fractions were used for immunoblotting. Western blot was performed based on instructions of LI-COR (LI-COR Corporation, Nebraska, USA). Primary antibodies used were Anti-BAK (no. 06-536; Millipore) Anti-cyto c (no. 556433; BD Bioscience), Anti-HA (no. 9110; Abcam), Anti-mtHsp70 (no. MA3-028; Thermo Scientific), and Anti-prohibitin (no. ab28172; Abcam), anti-Bax (N-20, sc-493, SantaCruz), anti-MCL-1 (ADI-AAP-240, Enzo life sciences), anti-Bid (no. AF860, R&D systems), anti-Bim (no.2933, Cell Signaling), anti-Bcl-xL (no. 610211, BD transduction), anti-Actin (no.612656, BD transduction), anti-Calnexin (no. ADI-SPA-860, Enzo Life sciences). Detection of bands was performed on a LI-COR Odyssey scanner. ImageJ was used for quantification of the bands.

### Oligomerization assay

Oligomerization has been performed as described previously^27^. In brief, pellet fractions from “ΔΨ_m_ measurement and cyto c Release Assay” section were treated with 10 mM freshly prepared cross-linker, BMH, for 30 min and then reaction was stopped with 20 mM Tris (pH 7.5). Samples were lysed as described in western blot section.

### Statistics

Samples were run as duplicates or triplicates. All experiments were repeated at least three times. Statistical significance was calculated using two-way ANOVA.

## Electronic supplementary material

supplemental material
