# Supplementary material for: Mitochondrial fusion and Bid-mediated mitochondrial apoptosis are perturbed by alcohol with distinct dependence on its metabolism
Source: Cell Death Dis. 2018 Oct 9;9(10):1028. doi: 10.1038/s41419-018-1070-3 (PMC6177459; doi:10.1038/s41419-018-1070-3)
Supplement: Supplementary file 1 — supplemental material [file 41419_2018_1070_MOESM1_ESM.pdf]

**Supplementary materials**

# Mitochondrial fusion and Bid-mediated mitochondrial apoptosis are perturbed by alcohol with distinct dependence on its metabolism

Shamim Naghdi[*], William Slovinsky[*], Muniswamy Madesh, Emanuel Rubin, and György Hajnóczky

**Supplementary methods**

**ADH activity assay**

ADH activity was recorded fluorometrically as the generation of NADH from EtOH oxidation to acetaldehyde by alcohol dehydrogenase essentially as it has been described before[1]. Briefly, harvested cells were washed and resuspended in cold phosphate buffer saline (PBS) and then disrupted by a sonicator (Sonic Dismembrator model 500, Fisher scientific) with 35% power, three pulses each took 5 second and 45 second intervals. Samples were centrifuged at 60,000 g for 40 min. 150 μg protein from the supernatant was in final volume of 630 μl containing: 0.5M Tris-HCl, 0.01 M DTT, 90 mM NAD. In the absence or presence of EtOH, NADH fluorescence was monitored in a fluorometer (Delta RAM, PTI) using 340 nm excitation and 460 nm emissions.

**CYP2E1 activity assay**

CYP2E1 activity was determined as hydroxylation of p-nitrophenol (PNP) to 4-nitrocatechol (4-NC) as described before with minor modification[1, 2]. Briefly, cells were washed and re-suspended in PBS, permeabilized by 40 μg/ml saponin and centrifuged for 5 min at 12,000 g. Pellets (Membrane fractions) were resuspended in cold 0.1M $K_2HPO_4/KH_2PO_4$, pH 7.4. In total volume of 100 μl, 300 μg protein was added to the buffer containing: 100 mM $K_2HPO4/KH_2PO_4$, pH 7.4, 0.4 mM PNP. NADH (1 mM) was added for 15 and 60 min in 37°C in shaking water bath. Reaction stopped by 8% TCA, samples were centrifuged at 10,000 g for 5 min and supernatant was transferred to a 96 well plate. 10 μl NaOH 10 N was added to each well and absorbance at 540 nm was recorded. In parallel various blank reactions were generated including no cell extract or no PNP or no NADPH.

**Supplementary Figure legends**

**Figure S.1. ADH and CYP2E1 enzymes are active in VL-17A.**

A) Representative time course recording of changes in NADH in the cytosolic fraction of VI-7 (left panel) and VL-17A (right panel) cells. Arrows show the addition of 100mM EtOH. The presented data is normalized to the initial intensity ($F_0$). Black and red traces are control and EtOH-preexposed (100mM for 72hr) samples in order. Comparing VI-7 cell line, VL-17A cells generate NADH rapidly. B) Bar charts show the linear fit to the slope of NADH curves in A. C) Bar charts show 4-nitrocatechol (4-NC) in the VI-7 (left panel) samples and VL-17A (right panel) samples after 15min/1h treatment with PNP. Black and red bar charts are control and EtOH exposed cells. Reactions without NADPH, PNP and microsomes (open bar charts) have been used as controls. EtOH-treated VL-17A cells show significant CYP2E1 activity already detectable at 15min and progressively increasing until 1h treatment with PNP. Without EtOH-pretreatment VL-17A cells have lesser CYP2E1 activity that causes detectable 4-NC production after 1h but is only a small fraction of that by the EtOH-stimulated ones. D) Immunoblot against CYP2E1 in the membrane fraction of zero and 100mM ethanol exposed VI-7 and VL-17A cells. Membrane fraction of mouse hepatocyte was used as positive control. Calnexin, Prohibitin and cyto c have been used as loading control. Western blot shows a light band for CYP2E1 in VL-17A cells which is greatly increased by EtOH pre-treatment (n=2) *: $p<0.05$.

**Figure S.2. HepG2 and VI-7 cells show similar mtPA-GFP spreading kinetic. EtOH metabolism inhibitors abolishe the effect of EtOH in VL-17A cells.**

A) Cells were transfected with mitochondrial matrix targeted DsRed (mtDsRed) and mtPA-GFP. Mitochondrial morphology and GFP spreading after photoactivation in designated areas is shown for a typical HepG2 (upper panel) and VI-7 cells (lower panel). B) Mean traces of time lapse of mtPA-GFP spreading after photoactivation that presented as normalized ratio of PA-GFP to mtDsRed in photo bleached area in HepG2 and VI-7. N was 39 and 33 for HepG2 and VI-7, respectively (n=3). C) Each bar represents mean±SE value of difference in mtPA-GFP spreading between control and EtOH treated cells in the presence or absence of MP-4 or ALDA-1 at 156 second (re-generated from Figure 2). D) Similar to Figure 3B except that EtOH treatment is acute.

N was 62 and 65 for untreated and acutely treated HepG2 cells and 13 and 23 for untreated and acutely treated VL-17A. E) Similar to Figure 3C except that EtOH treatment is acute. N was 74 and 105 for untreated and acutely treated HepG2 cells. N was 20 and 29 for untreated and acutely treated VL-17A. *: $p<0.05$.

**Figure S.3. Quantification of mitochondrial fusion proteins: Mfn1/2 and OPA1 shows no difference in the EtOH and mock-treated cells.**
Each bar chart represent the mean value of integrated intensity of relevant western blot band normalized to the intensity of Actin used as a loading control (n=3).

**Figure S.4. Purity of membrane and cytosolic fraction were tested. tBid-induced OMMP leads to Smac release to cytosol and it is augmented in EtOH-pretreated cells. Ethanol sensitized H9c2 cell, to tBid induced OMMP.**
A) Western blot of membrane and cytosolic fraction of the permeabilized cells that were separated quickly for mitochondrial HSP70, Prohibitin and Tubulin as mitochondria and cytosolic markers. B) Western blot of the cytosolic fraction of zero and 100 mM ethanol exposed HepG2 cells using Anti-Smac. Samples were treated for 300s with 0.5, 2.5 or 100 nM tBid. C) Time course recording of $\Delta\Psi_m$ in the permeabilized H9c2 cell suspension that were exposed to 40 or 80 mM ethanol. tBid (0 or 2.5 nM) was added at 500s. Arrow shows the tBid addition. D) The same as C except tBid concentration was zero or 0.032 nM (n=3). E) Western blot of cyto c in cytosolic parts which were treated with tBid (0, 0.0032 or 2.5 nM).

**Figure S.5. Quantification of western blots bands for Bax, $Bim_{EL}$, $Bim_L$, $Bim_S$ and endogenous tBid.** Intensity of the relevant band for Bax, $Bim_{EL}$, $Bim_L$, $Bim_S$ and endogenous tBid were normalized to the Actin.

## Supplementary references:


1. Osna NA, Clemens DL, Donohue TM, Jr. Interferon gamma enhances proteasome activity in recombinant Hep G2 cells that express cytochrome P4502E1: modulation by ethanol. *Biochemical pharmacology* 2003, **66**(5)**:** 697-710.

2. Chen Q, Galleano M, Cederbaum AI. Cytotoxicity and apoptosis produced by arachidonic acid in HepG2 cells overexpressing human cytochrome P-4502E1. *Alcoholism, clinical and experimental research* 1998, **22**(4)**:** 782-784.

Figure S1

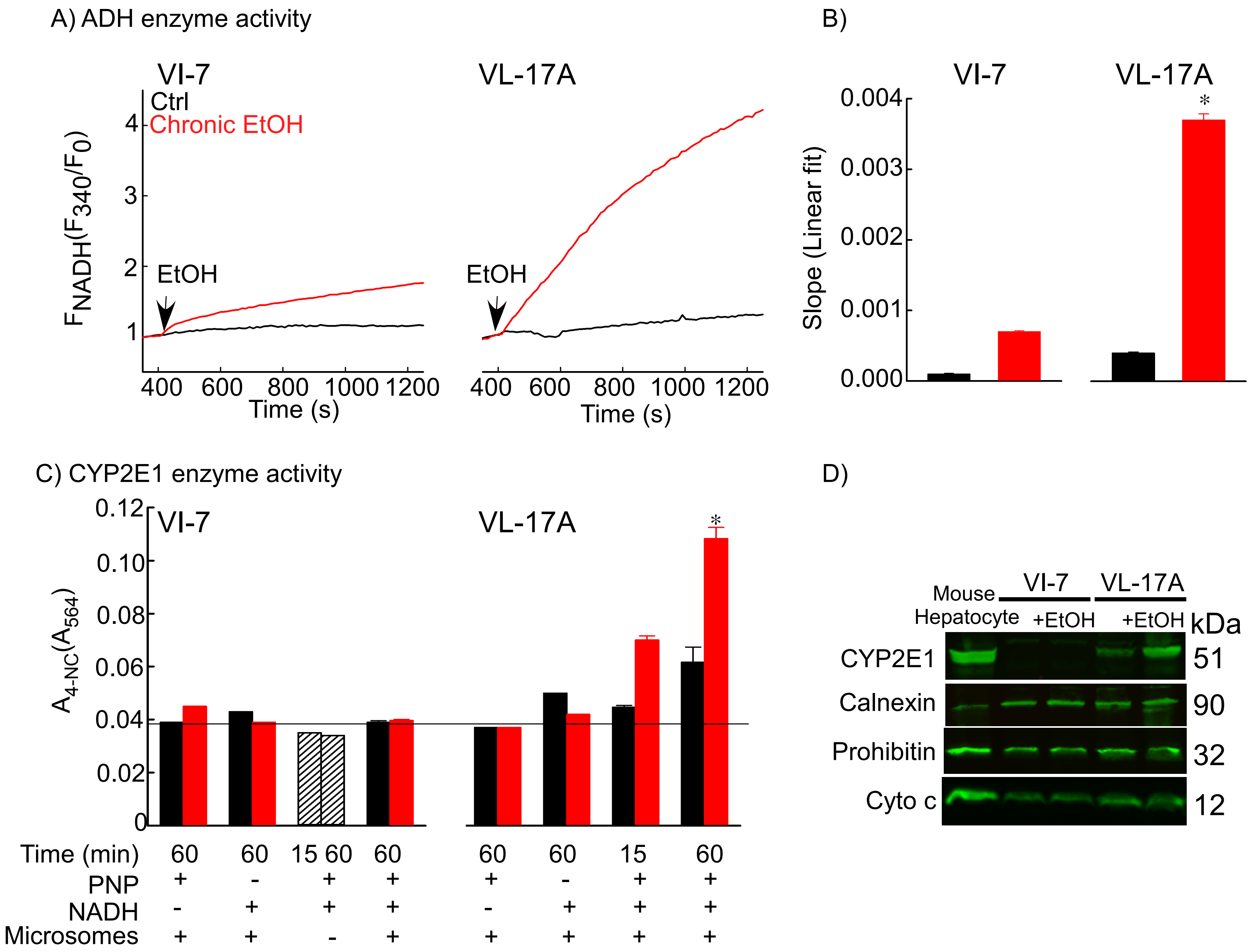
A) ADH enzyme activity
VI-7
VL-17A
Ctrl
Chronic EtOH
EtOH
$F_{NADH}(F_{340}/F_0)$
Time (s)
B)
VI-7
VL-17A
Slope (Linear fit)
*
C) CYP2E1 enzyme activity
VI-7
VL-17A
$A_{4\text{-}NC}(A_{564})$
Time (min)
PNP
NADH
Microsomes
D)
Mouse Hepatocyte
VI-7
VL-17A
+EtOH
+EtOH
kDa
CYP2E1
Calnexin
Prohibitin
Cyto c
51
90
32
12

Figure S2

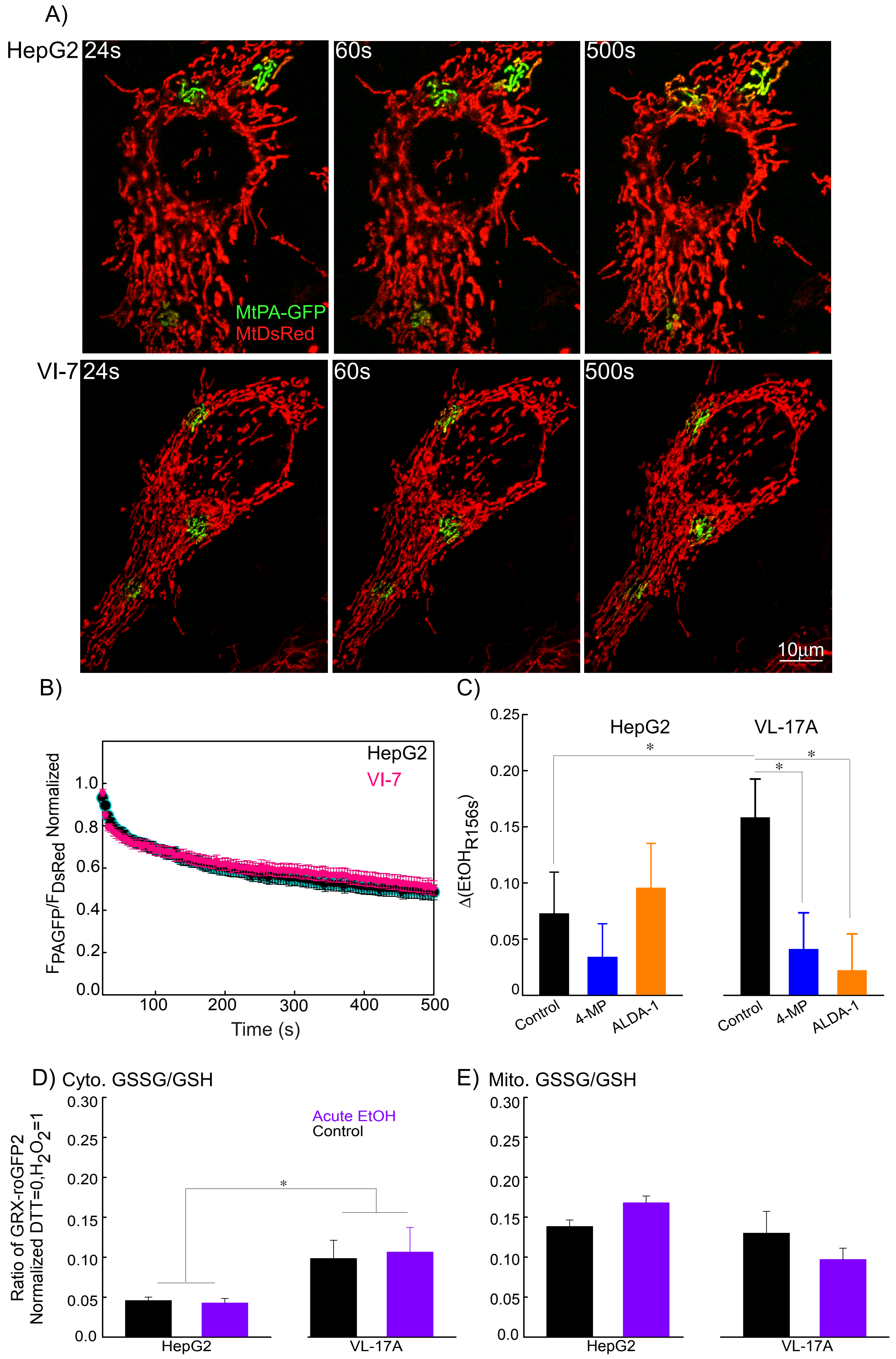

A)
HepG2 24s
60s
500s
MtPA-GFP
MtDsRed
VI-7 24s
60s
500s
10μm
B)
HepG2
VI-7
$F_{PAGFP}/F_{DsRed}$ Normalized
1.0
0.8
0.6
0.4
0.2
0.0
100
200
300
400
500
Time (s)
C)
HepG2
VL-17A
$\Delta(EtOH_{R156s})$
0.25
0.20
0.15
0.10
0.05
0
*
*
*
Control
4-MP
ALDA-1
Control
4-MP
ALDA-1
D) Cyto. GSSG/GSH
Acute EtOH
Control
Ratio of GRX-roGFP2
Normalized DTT=0,$H_2O_2$=1
0.30
0.25
0.20
0.15
0.10
0.05
0.0
*
HepG2
VL-17A
E) Mito. GSSG/GSH
0.30
0.25
0.20
0.15
0.10
0.05
0.0
HepG2
VL-17A

Figure S3

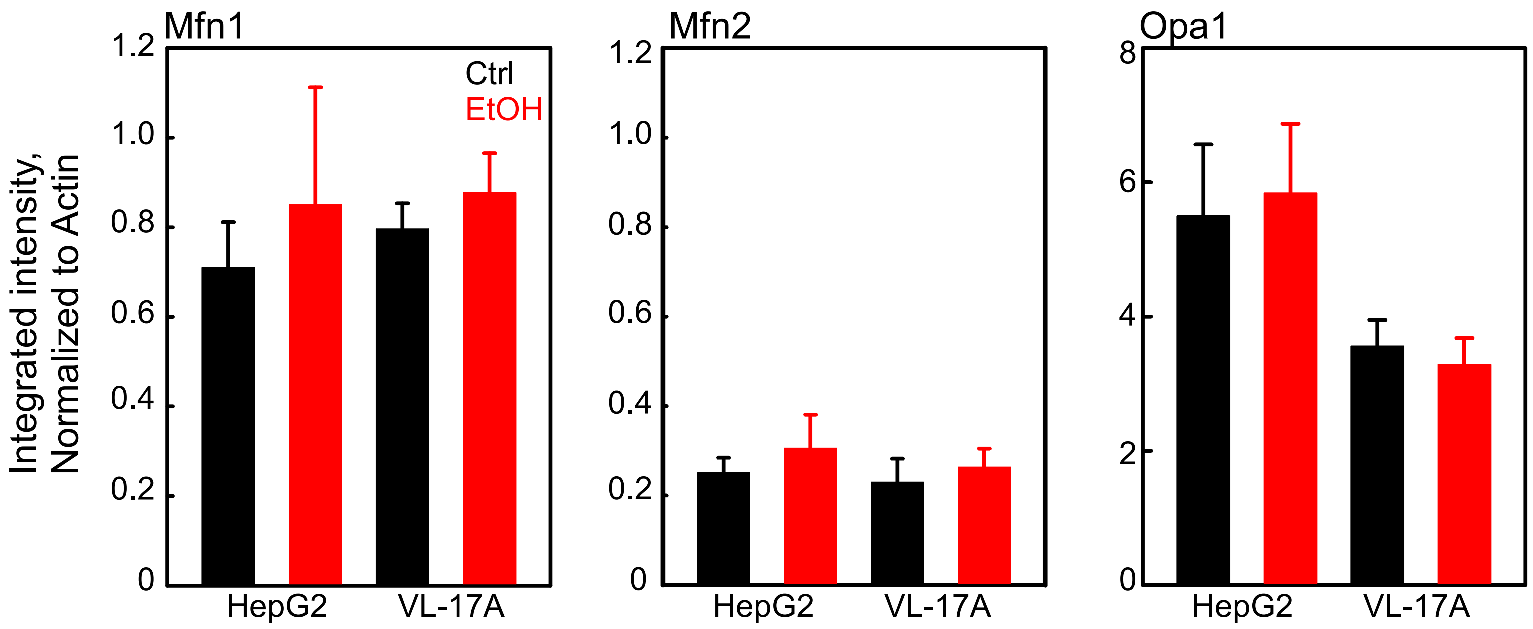
Mfn1
Mfn2
Opa1
Integrated intensity, Normalized to Actin
1.2
1.0
0.8
0.6
0.4
0.2
0
8
6
4
2
Ctrl
EtOH
HepG2
VL-17A

Figure S4

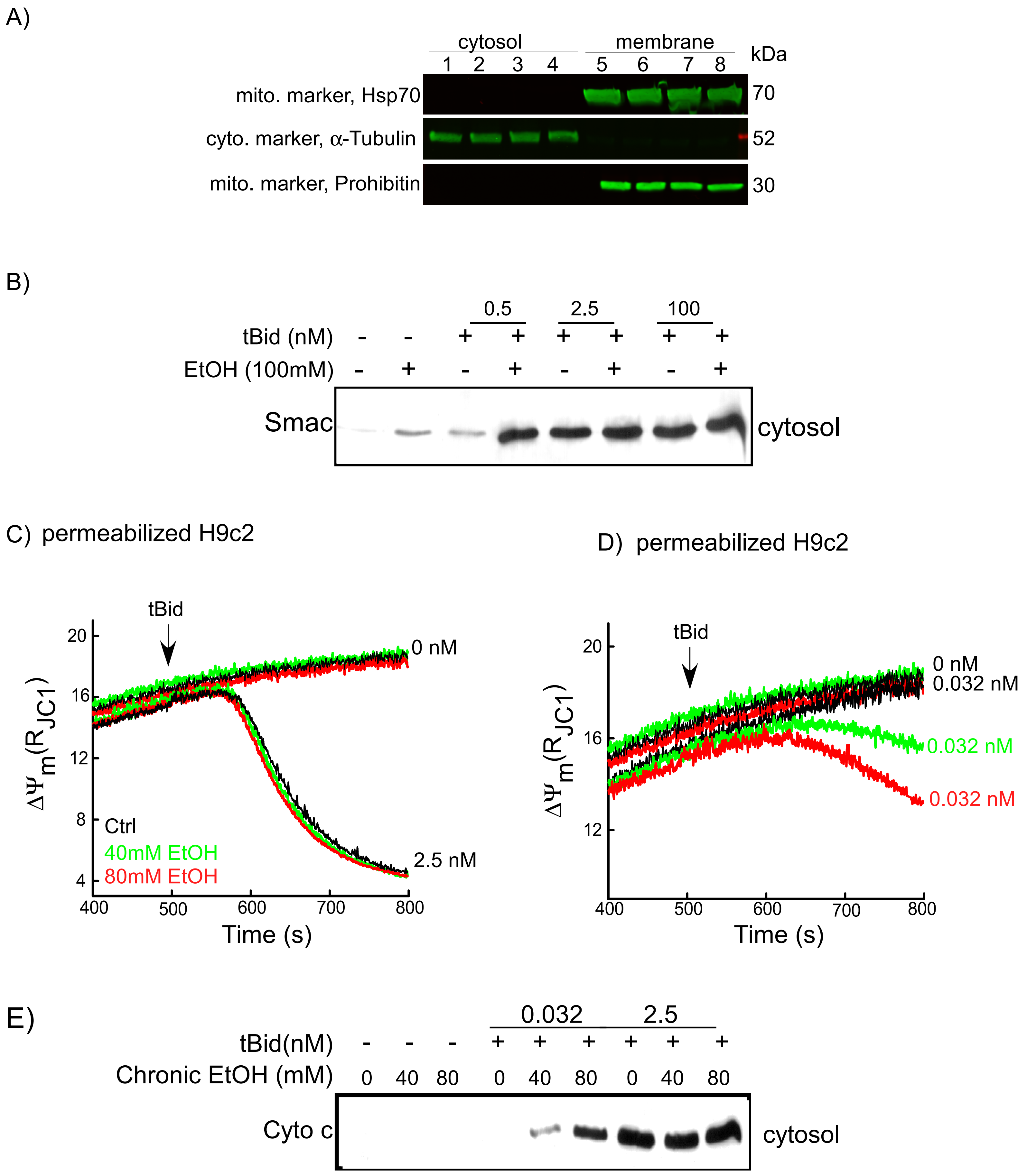

A)
cytosol
membrane
1 2 3 4 5 6 7 8
kDa
mito. marker, Hsp70
70
cyto. marker, α-Tubulin
52
mito. marker, Prohibitin
30
B)
0.5
2.5
100
tBid (nM)
EtOH (100mM)
Smac
cytosol
C) permeabilized H9c2
tBid
0 nM
2.5 nM
Ctrl
40mM EtOH
80mM EtOH
Time (s)
D) permeabilized H9c2
tBid
0 nM
0.032 nM
0.032 nM
0.032 nM
Time (s)
E)
0.032
2.5
tBid(nM)
Chronic EtOH (mM)
0 40 80 0 40 80 0 40 80
Cyto c
cytosol

Figure S5

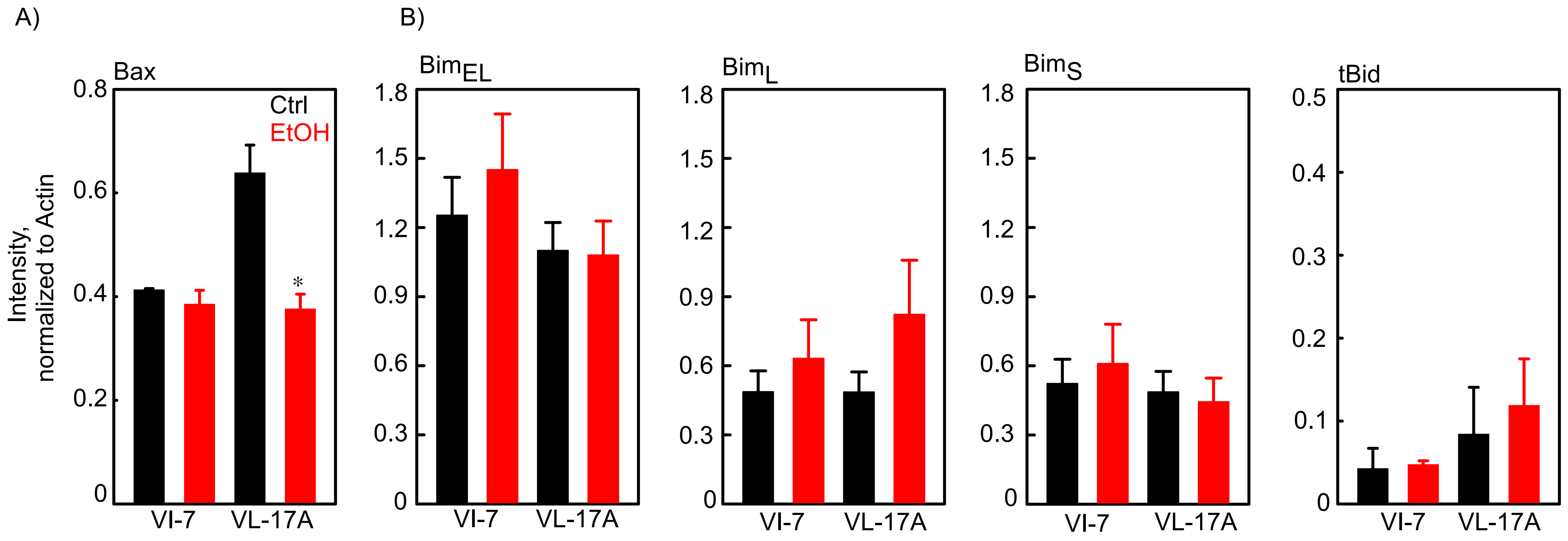

A)
B)
Bax
Ctrl
EtOH
Intensity, normalized to Actin
0.8
0.6
0.4
0.2
0
*
VI-7
VL-17A
Bim$_{EL}$
Bim$_{L}$
Bim$_{S}$
tBid
1.8
1.5
1.2
0.9
0.6
0.3
0.5
0.4
0.3
0.2
0.1
